# Identification of key genes involved in tumor immune cell infiltration and cetuximab resistance in colorectal cancer

**DOI:** 10.1186/s12935-021-01829-8

**Published:** 2021-02-25

**Authors:** Li Liang, Mengling Liu, Xun Sun, Yitao Yuan, Ke Peng, Khalid Rashid, Yiyi Yu, Yuehong Cui, Yanjie Chen, Tianshu Liu

**Affiliations:** 1 Department of Medical Oncology, Zhongshan Hospital, Fudan University, No. 180, Fenglin Road, Xuhui District, Shanghai, 200032 People’s Republic of China; 2 Department of Gastroenterology, Zhongshan Hospital, Fudan University, No. 180, Fenglin Road, Xuhui District, Shanghai, 200032 People’s Republic of China; 3 Center of Evidence-based Medicine, Fudan University, Shanghai, China; 4 Shanghai Institute of Liver Diseases, Shanghai, China

**Keywords:** Colorectal cancer, Drug resistance, Anti‐epidermal growth factor receptor therapy, Tumor immune cell infiltration, Transcriptional alterations

## Abstract

**Background:**

The anti-epidermal growth factor receptor (EGFR) antibody introduces adaptable variations to the transcriptome and triggers tumor immune infiltration, resulting in colorectal cancer (CRC) treatment resistance. We intended to identify genes that play essential roles in cetuximab resistance and tumor immune cell infiltration.

**Methods:**

A cetuximab-resistant CACO2 cellular model was established, and its transcriptome variations were detected by microarray. Meanwhile, public data from the Gene Expression Omnibus and The Cancer Genome Atlas (TCGA) database were downloaded. Integrated bioinformatics analysis was applied to detect differentially expressed genes (DEGs) between the cetuximab-resistant and the cetuximab-sensitive groups. Then, we investigated correlations between DEGs and immune cell infiltration. The DEGs from bioinformatics analysis were further validated in vitro and in clinical samples.

**Results:**

We identified 732 upregulated and 1259 downregulated DEGs in the induced cellular model. Gene Ontology and Kyoto Encyclopedia of Genes and Genomes pathway enrichment analyses, along with Gene Set Enrichment Analysis and Gene Set Variation Analysis, indicated the functions of the DEGs. Together with GSE59857 and GSE5841, 12 common DEGs (*SATB-2*, *AKR1B10*, *ADH1A*, *ADH1C*, *MYB*, *ATP10B*, *CDX-2*, *FAR2*, *EPHB2*, *SLC26A*3, *ORP-1*, *VAV*3) were identified and their predictive values of cetuximab treatment were validated in GSE56386. In online Genomics of Drug Sensitivity in Cancer (GDSC) database, nine of twelve DEGs were recognized in the protein-protein (PPI) network. Based on the transcriptome profiles of CRC samples in TCGA and using Tumor Immune Estimation Resource Version 2.0, we bioinformatically determined that *SATB-2, ORP-1, MYB*, and *CDX-2* expressions were associated with intensive infiltration of B cell, CD4^+^ T cell, CD8^+^ T cell and macrophage, which was then validated the correlation in clinical samples by immunohistochemistry. We found that *SATB-2*, *ORP-1, MYB*, and *CDX-2* were downregulated in vitro with cetuximab treatment. Clinically, patients with advanced CRC and high *ORP-1* expression exhibited a longer progression-free survival time when they were treated with anti-EGFR therapy than those with low *ORP-1* expression.

**Conclusions:**

*SATB-2, ORP-1, MYB*, and *CDX-2* were related to cetuximab sensitivity as well as enhanced tumor immune cell infiltration in patients with CRC.

## Background

In the United States, colorectal cancer (CRC) is estimated to be the third-most frequently occurring malignant tumor and third leading cause of cancer-related mortality, leading to the deaths of about 53,200 people in 2020 [1]. The incidence and mortality rates in 2015 were 37,000 cases and 19,000 deaths, with a 25- to 28-months overall survival time for Chinese patients suffering advanced CRC [2]. Targeting epidermal growth factor receptor (EGFR), monoclonal antibodies cetuximab and panitumumab are effective therapeutics [3, 4] that shrink tumors for resectability [5–7] or relieve symptoms from unresectable masses by repressing tumor growth [8–10].

Mutations in the *RAS* family suggest continuous activation of EGFR downstream signaling [11–15] and *RAS* genes (*KRAS*, *NRAS*) are used as biomarkers to predict poor response to anti-EGFR therapy [16, 17]. However, tumor microenvironment re-plasticity, characterized by tumor immune cell infiltration and upregulation of immune checkpoint-related proteins, has recently been recognized as a novel explanation for drug resistance and treatment failure [18–20]. In particular, the interaction between tumor and immune microenvironment triggered by cetuximab or panitumumab in CRC accounts for immunogenic cell death and immune treatment resistance [21, 22].

In this study, we firstly performed high-throughput screening of transcriptomic alterations before and after induction cetuximab administration in a colon cancer cell model (CACO2). Then, we conducted integrated bioinformatics analysis of the transcriptome variations using public datasets from the Gene Expression Omnibus (GEO) database to identify several core differentially expressed genes (DEGs) and investigated whether they were correlated with tumor immune cell infiltration. Finally, we validated the identified core DEGs in vitro and in clinical samples.

## Methods

### Cellular model and culture

Cell lines of human CRC (CACO2, HCT116, HT29, NCIH508, and RKO) were obtained from the Cell Bank of the Chinese Academy of Sciences (Shanghai, China). All cell lines were authenticated by short tandem repeat (STR) profiling (Genetic Testing Biotechnology Corporation, Suzhou, China) and routinely tested for mycoplasma using MycoAlert™ Mycoplasma Detection Kit (Lonza; LT07-218, Rockland, ME, USA). We established an EGFR antagonist-resistant cellular model using cetuximab sensitive cell line (CACO2-CS) and validated the transcriptional changes induced by cetuximab in the other cell lines. Using stepwise induction, we started with a low dose causing 50% cell growth inhibition (IC_50_). The dose was increased to 10 µg/mL after about two months, 50 µg/mL after another two months, and lastly 300 µg/mL. Cell Counting Kit 8 (CCK8) (Dojindo, Shanghai, China) was used to test the cell viability. For colonies formation assay, cells were cultured in six-well plates with 1000 cells and 2 ml media including cetuximab (300 µg/ml) per well. After 14 days, colonies were fixed with 4% paraformaldehyde and stained with 0.1% crystal violet. Finally, the established cetuximab resistant cell line (CACO2-CR) was cultured with the maximal dose of cetuximab. CACO2-CS, HCT116, HT29, NCIH508, and RKO in the exponential growth phase were precultured in 12-well tissue culture plates for 24 h. Different concentrations of cetuximab (S20130004, Merck KGaA, Darmstadt, Germany) were added to cells which were incubated for 72 h (0 µg/mL and 25 µg/mL for CACO2, 0 µg/mL and 1 µg/mL for NCIH508, and 0 µg/mL and 50 µg/mL for HT29, HCT116, and RKO). CACO2-CS and CACO2-CR cells were cultured in Dulbecco’s Modified Eagle’s Medium (DMEM) (HyClone, Logan, UT, USA) containing 20% fetal bovine serum (FBS) (Gibco, Paisley, UK) 100 U/mL penicillin, and 100 U/mL streptomycin (Gibco, Paisley, UK). HT-29 cells were cultured in McCoy’s 5A (Gibco, Grand Island, NY, USA) supplemented with 10% FBS (Gibco, Paisley, UK), 100 U/mL penicillin, and 100 U/mL streptomycin (Gibco, Paisley, UK). HCT116 and NCIH508 cells were cultured in Roswell Park Memorial Institute Modified Medium (Hyclone) supplemented with 10% FBS (Gibco, Paisley, UK), 100 U/mL penicillin, and 100 U/mL streptomycin (Gibco, Paisley, USA). RKO cells were cultured in high glucose DMEM (Hyclone) supplemented with 10 % FBS (Gibco, Paisley, UK), 100 U/mL penicillin, and 100 U/mL streptomycin (Gibco, Paisley, UK). All cells were cultured in a humidified 5% CO_2_ incubator at 37 °C. All cell lines experiments were repeated at least three times with three to six replicates.

### Microarray screening for transcriptional variation after cetuximab induction

Total RNA was extracted and purified with the miRNeasy Mini Kit (Cat. # 217,004 QIAGEN GmBH, Hilden, Germany) according to the manufacturer’s instructions. The RNA integrity number was determined with the Agilent Bioanalyzer 2100 (Agilent Technologies, Santa Clara, CA, USA), and the RNA was amplified and labeled with the Low Input Quick Amp WT Labeling Kit (Cat. # 5190−2943, Agilent Technologies, Santa Clara, CA, USA). Labeled cRNA was purified with the RNeasy mini kit (Cat. # 74,106). Samples were hybridized with about 1.65 µg Cy3-labeled cRNA by the Gene Expression Hybridization Kit (Cat. # 5188–5242, Agilent Technologies) in a hybridization oven (Cat. # G2545A, Agilent Technologies). After about 17 h, the slides were washed in staining dishes (Cat. # 121, Thermo Shandon, Waltham, MA, USA) with the Gene Expression Wash Buffer Kit (Cat. # 5188–5327, Agilent Technologies) and scanned by the Agilent Microarray Scanner (Cat. # G2565CA, Agilent Technologies). Data were retrieved with Feature Extraction software 10.7 (Agilent Technologies). Quantile normalization of the raw data was achieved by the limma package in R (version 3.4.1) [23].

### **Real-time quantitative PCR after cetuximab treatment** in vitro

Total cellular RNA of cell lines treated with cetuximab was extracted with TRIzol reagent (Invitrogen, Carlsbad, CA, USA) under the manufacturer’s instructions. Recombinant DNase I (Takara Bio, Beijing, China) was used to remove potential genomic DNA contamination. cDNA was generated with the PrimerScript™ RT master mix kit (Takara Bio, Dalian China). Real-time quantitative PCR analysis of the identified DEGs was performed using the TB Green Premix Ex Taq™ kit (Takara Bio, Dalian China). All results were normalized to human GAPDH mRNA expression. The primers were listed in Additional file 1: Table S1. The relative threshold cycle (Ct) method was used to display the results.

### Identification of differentially expressed genes (DEGs) in the cellular model

For the microarray screening of CACO2 cells treated with cetuximab, we visualized the principal component analysis (PCA) results using the R package ggbiplot (https://github.com/vqv/ggbiplot) after converting the raw signals into gene expression levels. We set thresholds of *p* < 0.05 and | log2 (fold change) | > 1 to identify significant DEGs using the limma package and created heat maps using the heatmap package.

### Gene expression omnibus (GEO) microarray data and DEGs analysis

To further narrow down candidate DEGs in CRC under EGFR antagonist pressure, we downloaded three gene expression datasets including cell lines and clinical tissue sample data from GEO (http://www.ncbi.nlm.nih.gov/geo). GSE59857 included the transcriptional and pharmacological profiles of 155 CRC cell lines. GSE5851 contained data from 80 clinical advanced CRC samples obtained before cetuximab monotherapy. GSE56386 contained data from eight primary CRC tumor tissue samples, comprising four from responders to cetuximab therapy and four from non-responders. From GSE59857, we downloaded the data for 20 cell lines, including 10 cetuximab-sensitive cases (OXCO2: GSM1448146, NCIH508: GSM1448142, DIFI: GSM1448175, COCM1: GSM1448167, CCK81: GSM1448097, C75: GSM1448201, HCA46: GSM1448177, C99: GSM1448204, HDC82: GSM1448128, and COGA1: GSM1448099) and 10 cetuximab-insensitive cases (SNU1047: GSM1448085, COLO320DM: GSM1448173, HUTU80: GSM1448180, KM12: GSM1448073, HDC143: GSM1448185, KM12SM: GSM1448188, KM12C: GSM1448186, COLO320: GSM1448152, KM12L4: GSM1448187, and C10: GSM1448196), among which HDC143 was deemed invalid and removed after analysis. We then converted the probe IDs into gene symbols using illuminaHumanv4.db. We retrieved the data for eight patients with the wild-type *KRAS* gene, including four responders to cetuximab treatment (GSM136609, GSM136593, GSM136654, and GSM136626) and four non-responders (GSM136635, GSM136646, GSM136607, and GSM136640) from GSE5851 and converted the probe IDs into gene symbols using the annotation library hgu133a2.db. The procedure for analyzing DEGs was the same as that for analyzing the CACO2 microarray data.

### Gene Ontology (GO) term and Kyoto Encyclopedia of genes and genomes (KEGG) pathway enrichment analyses

For the enrichment analyses of the DEGs, a Metascape (https://metascape.org/) online analysis tool was used [24]. The parameters were set as the following: min overlap = 3, *p*-value cutoff = 0.01, and min enrichment = 1.5. Related terms were selected from the top 20, according to their *p*-values.

### Gene sets Enrichment analysis (GSEA) and Gene set variation analysis (GSVA)

GSEA was used to screen the biological states and processes associated with significantly upregulated or downregulated genes in the resistance group, with H.all.v7.1.symbols.gmt as the reference set [25]. The number of permutations was set to 1000. A *p*-value < 0.05 was considered indicative of significant enrichment. A normalized enrichment score was established to evaluate the degree of enrichment. GSVA was used to describe the enrichment degree of a biological state or process in a sample [26]. Enrichment scores were compared, and those meeting the standards of |log_2_ (fold change) | > 1 and *p* < 0.05 were considered significant results.

### Construction of protein‐protein interaction (PPI) network

The PPIs of the central DEGs were generated using the STRING online database (https://string-db.org). To construct a PPI network, we retrieved cetuximab resistance candidates from the Genomics of Drug Sensitivity in Cancer (GDSC) database (https://www.cancerrxgene.org) [27], confirmed their interactions using the STRING database [28], and finally visualized the result using Cystoscape [29].

### Prediction of tumor immune cell infiltration

To investigate the relationship between hub DEG expression and tumor immune cell infiltration, the transcriptome landscapes of colonic or rectal adenocarcinoma samples (n = 177) in The Cancer Genome Atlas (TCGA) database were analyzed using the online tool Tumor Immune Estimation Resource Version 2.0 (https://cistrome.shinyapps.io/timer/) [30, 31].

### Validation in clinical samples by immunohistochemistry staining

Patients with advanced CRC (*n* = 102) were diagnosed by pathological examination and administered systemic chemotherapeutics at Zhongshan Hospital of Fudan University. Fifty-two patients received therapy containing cetuximab. The progression-free survival time (PFS) was recorded to evaluate the efficacy of cetuximab. Patients signed informed consent documents, and the ethics committee of Zhongshan Hospital approved the study.

Tumor samples were fixed with 4% paraformaldehyde, embedded in paraffin, cut into sections of about 5 µm, and placed onto glass slides. After the samples were deparaffinized with xylene, hydrophilized, and unmasked, they were blocked with bovine serum albumin, immunostained with primary antibodies against *SATB-2* (21307-1-AP, 1:100, Proteintech Group, Inc., Wuhan, China), *ORP-1* (bs-17514R, 1:200, Bioss Biological Technology Co., Ltd., Beijing, China), *MYB* (bs-5978R, 1:200, Bioss Biological Technology Co., Ltd.), *CDX-2* (bsm-33063m, 1:200, Bioss Biological Technology Co., Ltd.), CD8 (GB13068, 1:100, Servicebio Technology, Wuhan, China), CD19 (GB11061, 1:500, Servicebio Technology), CD4 (GB13064, 1:100, Servicebio Technology), and CD68 (GB13067-M-2, 1:100, Servicebio Technology) in a humidified environment at about 4 °C overnight and incubated with goat anti-rabbit or anti-mouse secondary antibody (1:200) for about 30 min at about 20 °C. Subsequently, the slides were stained with 3,3’-diaminobenzidine and counterstained with hematoxylin. Antigen–antibody complexes in the whole sample were detected using a panoramic slice scanner (3DHISTECH, Budapest, Hungary), recorded in a file, and viewed using CaseViewer 2.2 (3DHISTECH). To evaluate gene expression in tissues, the following formula was used to calculate the H-score using Quant Center 2.1 (3DHISTECH): H-SCORE = ∑ (PI × I) = (percentage of cells of weak intensity × 1) + (percentage of cells of moderate intensity × 2) + percentage of cells of strong intensity × 3), where PI is the proportion of the positive signal pixel area and I is the coloring intensity.

### Statistical analysis

Most statistical analyses were completed using bioinformatic tools mentioned above in *R* (version 3.4.1). The Benjamini and Hochberg False Discovery Rate method was utilized to adjust the *p-* values in screening DEGs from GEO profiles. Fisher’s exact test was employed to identify the significant GO terms and KEGG pathways. The correlation significance was examined by Spearman and Pearson correlation analyses. Differential expression levels of identified DEGs were assessed by a two-tailed Student’s *t*-test. Kaplan - Meier survival curve and Log - rank test analysis was applied to investigated the predictive valued of identified DEGs for patients with CRC and receiving cetuximab contained therapy. The value of *p* < 0.050 was considered statistically significant.

## Results

### Model establishment and screening of DEGs

**Fig. 1:**
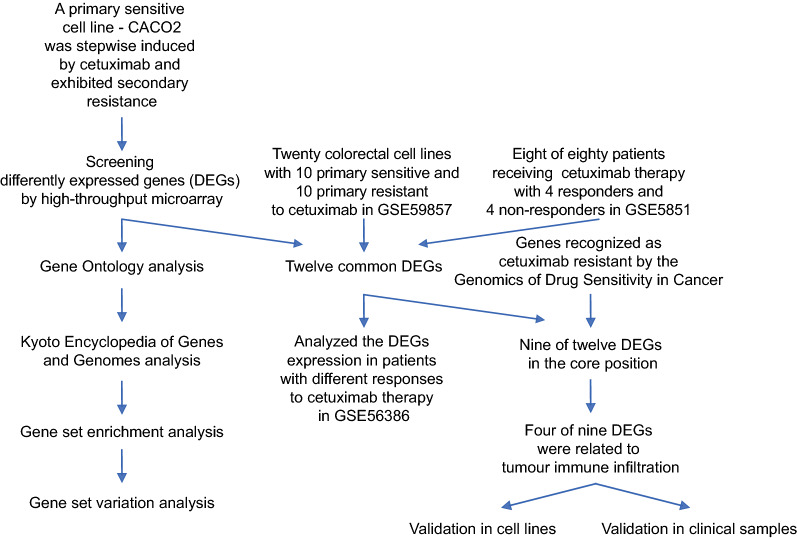
Flow chart of the study

**Fig. 2:**
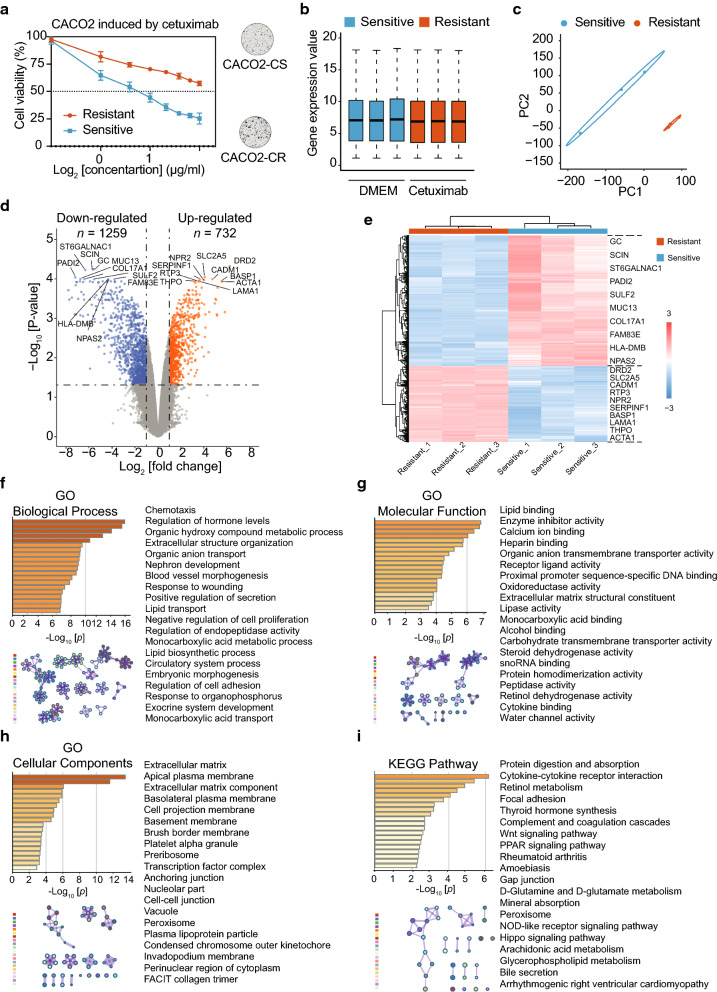
Identification and enrichment analysis of DEGs related to anti-EGFR antibody resistance in CACO2.** a** Establishment of a cetuximab-resistant CACO2 cell line (CACO2-CR) from its parental sensitive cell line (CACO2-CS) and validation of the resistance using cell viability tests. **b** Comparison of total gene expression between duplicate samples from the innate sensitive group (blue) and adaptive resistant (red) group. **c** Principal component analysis results suggested favorable reproducibility between the sensitive (blue) and resistant (red) groups. **d** The volcanic map reveals the distributions of downregulated (blue) and upregulated (orange) genes in the resistant group versus the sensitive group. Top 10 of DEGs between two groups were shown. **e** The heat map indicates the upregulated (red) and downregulated (blue) DEGs between the sensitive (blue) and resistant (red) groups. Each column is a sample and each row is a gene. Top 10 of DEGs between two groups were shown. **f** GO biological process term enrichment analysis of DEGs (left) and their interrelationships (right). **g** GO molecular function term enrichment analysis of DEGs (left) and their interrelationships (right). **h** GO cellular component term enrichment analysis of DEGs (left) and their interrelationships (right). **i** KEGG pathway enrichment analysis of DEGs (left) and their interrelationships (right). DEGs: Differentially expressed genes. GO: Gene Ontology. KEGG: Kyoto Encyclopedia of Genes and Genomes

The study design is shown in Fig. 1. After the cetuximab resistant cellular model was established successfully (Fig. 2a), preprocessing of the raw data revealed a uniform distribution of DEGs between CACO2-CS and CACO2-CR (Fig. 2b). The PCA results had acceptable reproducibility (Fig. 2c). A total of 1991 DEGs were identified, with 732 upregulated genes and 1259 downregulated genes in CACO2-CR. These DEGs are shown in a volcano plot (Fig. 2d) and a heat map (Fig. 2e) and are listed in Table 1. In GO analysis, the most enriched GO terms determined by the cellular component function (CC) were “extracellular matrix,” “apical plasma membrane” and “extracellular matrix component” (Fig. 2h). KEGG signaling analysis suggested that the DEGs were considerably enriched in “protein digestion and absorption,” “cytokine-cytokine receptor interaction,” “retinol metabolism” shown in Fig. 2i and Table 2.

**Table 1:** The expression of the top 20 upregulated and downregulated genes in the cetuximab resistant CACO2

Gene symbol	Normalized Signal Value						Log [fold change]	*P* value	Adjustive *p* value
	Sensitive			Resistant					
	Sam.1	Sam.2	Sam.3	Sam.1	Sam.2	Sam.3			
Top 20 of up-regulated genes									
*DRD2*	1.253	1.265	1.252	7.414	7.471	7.331	6.149	2.76E–09	5.19E–05
*SLC2A5*	8.931	9.006	9.037	13.002	12.996	12.980	4.001	2.62E–08	9.87E–05
*CADM1*	4.659	4.421	4.556	9.133	9.344	9.152	4.664	4.91E–08	1.11E–04
*RTP3*	1.232	1.300	1.161	4.731	4.881	4.823	3.581	8.34E–08	1.14E–04
*NPR2*	6.365	6.349	6.191	10.112	10.297	10.310	3.938	9.25E–08	1.16E–04
*SERPINF1*	10.281	10.168	10.135	13.543	13.716	13.685	3.453	1.29E–07	1.26E–04
*BASP1*	6.890	6.446	6.407	11.922	12.156	12.069	5.468	1.33E–07	1.26E–04
*LAMA1*	6.237	6.392	6.334	9.733	9.873	9.949	3.531	1.42E–07	1.26E–04
*THPO*	1.271	1.275	1.418	4.485	4.429	4.562	3.171	1.75E–07	1.26E–04
*ACTA1*	4.330	3.837	4.273	9.829	9.596	9.503	5.496	1.80E–07	1.26E–04
*SERPINF2*	11.096	11.257	10.992	14.287	14.346	14.294	3.194	2.59E–07	1.35E–04
*ANXA8*	9.699	9.805	9.662	12.950	13.168	12.871	3.274	3.77E–07	1.73E–04
*DPYSL3*	7.688	7.165	7.810	12.588	12.744	12.676	5.115	4.15E–07	1.82E–04
*PDGFRA*	1.312	1.304	1.231	5.091	5.216	4.782	3.748	4.25E–07	1.82E–04
*SDC2*	4.645	4.510	4.744	7.533	7.547	7.725	2.968	5.37E–07	2.10E–04
*ORM1*	12.007	12.089	11.909	14.634	14.613	14.758	2.667	5.43E–07	2.10E–04
*ORM2*	12.062	12.209	12.006	14.728	14.646	14.739	2.612	5.85E–07	2.10E–04
*ADM*	10.696	10.620	10.518	13.201	13.321	13.175	2.621	5.92E–07	2.10E–04
*ADAM19*	9.666	9.526	9.323	12.874	12.710	12.676	3.248	6.38E–07	2.22E–04
*CCDC3*	9.139	9.112	8.781	12.448	12.206	12.351	3.324	9.05E–07	2.59E–04
Top 20 of down-regulated genes									
*GC*	6.719	6.693	6.902	1.271	1.278	1.263	–5.500	8.90E–09	6.23E–05
*SCIN*	6.839	6.926	7.093	1.350	1.309	1.335	–5.621	9.94E–09	6.23E–05
*ST6GALNAC1*	7.759	7.546	7.389	1.269	1.309	1.356	–6.253	1.39E–08	6.51E–05
*PADI2*	8.533	8.655	8.104	1.339	1.256	1.373	–7.108	3.18E–08	9.95E–05
*MUC13*	14.179	13.905	14.478	7.486	7.521	7.534	–6.674	4.05E–08	1.09E–04
*SULF2*	9.285	8.985	9.273	3.737	3.522	3.850	–5.478	5.92E–08	1.11E–04
*COL17A1*	8.492	8.749	8.525	2.545	2.030	2.235	–6.319	6.07E–08	1.11E–04
*FAM83E*	10.754	10.695	10.635	6.944	7.056	6.897	–3.729	6.48E–08	1.11E–04
*HLA-DMB*	5.666	5.548	5.337	1.177	1.237	1.244	–4.298	8.23E–08	1.14E–04
*NPAS2*	5.506	5.310	5.640	1.250	1.186	1.173	–4.282	8.47E–08	1.14E–04
*PNLIPRP2*	9.009	8.923	8.898	4.505	4.836	4.560	–4.310	1.04E–07	1.22E–04
*LCN2*	11.287	11.659	11.027	4.564	4.442	4.834	–6.711	1.24E–07	1.26E–04
*SRPX*	9.803	9.612	9.507	5.937	5.942	5.842	–3.734	1.53E–07	1.26E–04
*HOXB9*	10.762	10.582	10.682	7.503	7.485	7.573	–3.155	1.66E–07	1.26E–04
*BTNL3*	5.120	5.170	5.485	1.188	1.231	1.215	–4.047	1.77E–07	1.26E–04
*TMC5*	7.201	6.746	7.401	1.274	1.203	1.339	–5.844	1.77E–07	1.26E–04
*APOBEC1*	6.060	5.632	6.092	1.210	1.170	1.156	–4.749	1.78E–07	1.26E–04
*S100A9*	10.469	10.404	9.896	3.540	3.948	3.972	–6.436	2.00E–07	1.31E–04
*DDIT4L*	6.599	6.789	7.249	1.229	1.281	1.306	–5.607	2.07E–07	1.31E–04
*PI3*	13.824	13.941	13.611	9.571	9.226	9.378	–4.400	2.12E–07	1.31E–04

**Table 2:** GO term and KEGG pathway enrichment analyses of differentially expressed genes

Term	Description	Count	%	Log_10_ [*p* value]	Log_10_ [*q* value]
GO Biological process					
GO:0006935	Chemotaxis	117	5.89	−15.44	−11.44
GO:0010817	Regulation of hormone levels	102	5.13	−15.05	−11.33
GO:1,901,615	Organic hydroxy compound metabolic process	100	5.03	−13.61	−10.01
GO:0043062	Extracellular structure organization	82	4.13	−12.37	−8.87
GO:0015711	Organic anion transport	85	4.28	−10.60	−7.25
GO:0072006	Nephron development	37	1.86	−9.56	−6.40
GO:0048514	Blood vessel morphogenesis	105	5.28	−9.29	−6.20
GO:0009611	Response to wounding	105	5.28	−9.25	−6.20
GO:0051047	Positive regulation of secretion	75	3.77	−9.14	−6.11
GO:0006869	Lipid transport	66	3.32	−9.03	−6.06
GO:0008285	Negative regulation of cell proliferation	113	5.69	−8.91	−6.01
GO:0052548	Regulation of endopeptidase activity	73	3.67	−8.70	−5.86
GO:0032787	Monocarboxylic acid metabolic process	97	4.88	−8.15	−5.36
GO:0008610	Lipid biosynthetic process	103	5.18	−7.83	−5.11
GO:0003013	Circulatory system process	83	4.18	−7.17	−4.54
GO:0048598	Embryonic morphogenesis	87	4.38	−6.84	−4.26
GO:0030155	Regulation of cell adhesion	99	4.98	−6.68	−4.14
GO:0046683	Response to organophosphorus	31	1.56	−6.68	−4.14
GO:0035272	Exocrine system development	17	0.86	−6.62	−4.11
GO:0015718	Monocarboxylic acid transport	35	1.76	−6.53	−4.04
GO molecular function					
GO:0008289	Lipid binding	104	5.23	−6.90	−3.45
GO:0004857	Enzyme inhibitor activity	62	3.12	−6.81	−3.45
GO:0005509	Calcium ion binding	97	4.88	−6.45	−3.37
GO:0008201	Heparin binding	34	1.71	−6.05	−3.17
GO:0008514	Organic anion transmembrane transporter activity	39	1.96	−5.75	−3.06
GO:0048018	Receptor ligand activity	70	3.52	−5.72	−3.06
GO:0000987	Proximal promoter sequence-specific DNA binding	84	4.23	−5.16	−2.65
GO:0016491	Oxidoreductase activity	95	4.78	−4.82	−2.41
GO:0005201	Extracellular matrix structural constituent	30	1.51	−4.52	−2.18
GO:0016298	Lipase activity	25	1.26	−4.46	−2.16
GO:0033293	Monocarboxylic acid binding	18	0.91	−4.39	−2.14
GO:0043178	Alcohol binding	19	0.96	−4.39	−2.14
GO:0015144	Carbohydrate transmembrane transporter activity	12	0.60	−4.35	−2.12
GO:0033764	Steroid dehydrogenase activity	10	0.50	−4.05	−1.90
GO:0030515	snoRNA binding	10	0.50	−4.05	−1.90
GO:0042803	Protein homodimerization activity	81	4.08	−4.04	−1.90
GO:0008233	Peptidase activity	78	3.93	−3.82	−1.73
GO:0004745	Retinol dehydrogenase activity	8	0.40	−3.80	−1.73
GO:0019955	Cytokine binding	24	1.21	−3.69	−1.63
GO:0015250	Water channel activity	6	0.30	−3.50	−1.46
GO cellular component					
GO:0031012	Extracellular matrix	98	4.93	−13.46	−10.17
GO:0016324	Apical plasma membrane	68	3.42	−11.60	−8.79
GO:0044420	Extracellular matrix component	17	0.86	−6.08	−3.57
GO:0016323	Basolateral plasma membrane	41	2.06	−5.91	−3.52
GO:0031253	Cell projection membrane	56	2.82	−5.90	−3.52
GO:0005604	Basement membrane	23	1.16	−5.55	−3.21
GO:0031526	Brush border membrane	16	0.81	−5.25	−2.97
GO:0031091	Platelet alpha granule	21	1.06	−4.93	−2.72
GO:0030684	Pre-ribosome	19	0.96	−4.87	−2.70
GO:0005667	Transcription factor complex	53	2.67	−4.60	−2.46
GO:0070161	Anchoring junction	71	3.57	−3.64	−1.68
GO:0044452	Nucleolar part	29	1.46	−3.61	−1.67
GO:0005911	Cell-cell junction	53	2.67	−3.49	−1.60
GO:0005773	Vacuole	93	4.68	−3.40	−1.56
GO:0005777	Peroxisome	24	1.21	−3.40	−1.56
GO:0034358	Plasma lipoprotein particle	10	0.50	−3.32	−1.52
GO:0000940	Condensed chromosome outer kinetochore	6	0.30	−3.29	−1.52
GO:0071438	Invadopodium membrane	4	0.20	−3.22	−1.48
GO:0048471	Perinuclear region of cytoplasm	85	4.28	−3.20	−1.47
GO:0005593	FACIT collagen trimer	4	0.20	−2.88	−1.25
KEGG pathway					
hsa04974	Protein digestion and absorption	23	1.16	−6.16	−3.46
hsa04060	Cytokine-cytokine receptor interaction	45	2.26	−5.38	−2.98
hsa00830	Retinol metabolism	17	0.86	−4.86	−2.64
hsa04510	Focal adhesion	34	1.71	−4.45	−2.36
hsa04918	Thyroid hormone synthesis	17	0.86	−4.08	−2.08
hsa04610	Complement and coagulation cascades	17	0.86	−3.71	−1.85
hsa04310	Wnt signaling pathway	24	1.21	−3.21	−1.49
hsa03320	PPAR signaling pathway	15	0.75	−3.18	−1.49
hsa05323	Rheumatoid arthritis	17	0.86	−3.02	−1.36
hsa05146	Amoebiasis	17	0.86	−2.70	−1.27
hsa04540	Gap junction	16	0.81	−2.69	−1.27
hsa00471	D-Glutamine and D-glutamate metabolism	3	0.15	−2.68	−1.27
hsa04978	Mineral absorption	11	0.55	−2.60	−1.21
hsa04146	Peroxisome	15	0.75	−2.53	−1.16
hsa04621	NOD-like receptor signaling pathway	25	1.26	−2.49	−1.14
hsa04390	Hippo signaling pathway	23	1.16	−2.42	−1.13
hsa00590	Arachidonic acid metabolism	12	0.60	−2.38	−1.13
hsa00564	Glycerophospholipid metabolism	16	0.81	−2.35	−1.12
hsa04976	Bile secretion	13	0.65	−2.32	−1.10
hsa05412	Arrhythmogenic right ventricular cardiomyopathy	13	0.65	−2.26	−1.06

GO, Gene Ontology. KEGG, Kyoto Encyclopedia of Genes and Genomes

### Gene set enrichment

**Fig. 3:**
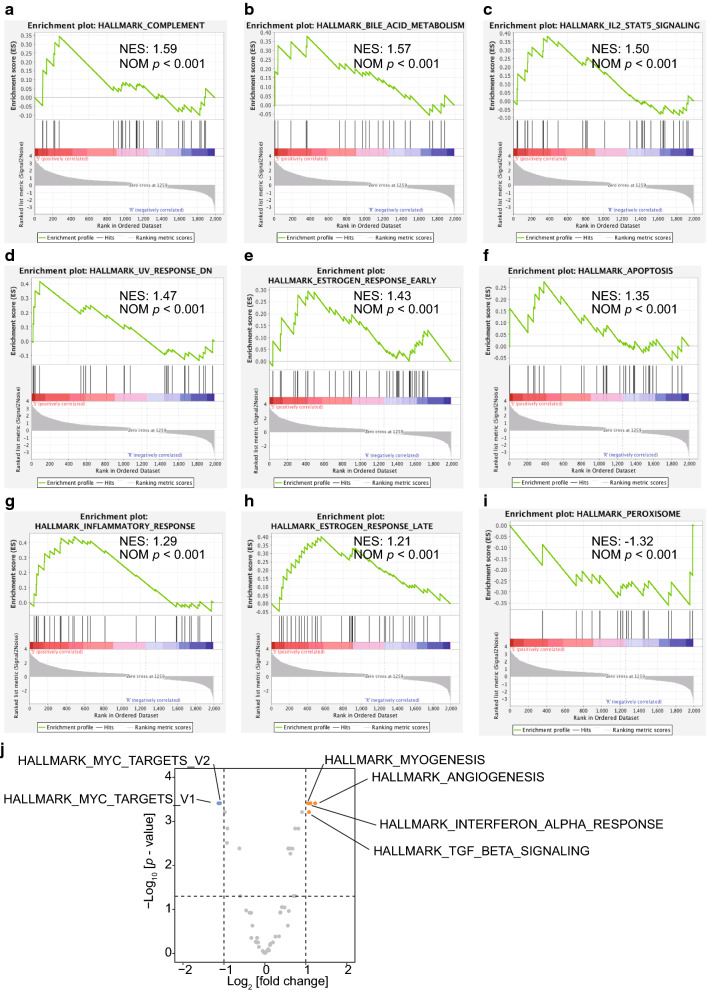
GSEA and GSVA of DEGs. **a-h** GSEA of downregulated and **(i)** upregulated DEGs involved in biological states and processes in the resistant group, compared with those in the sensitive group. **j** Volcanic diagram of the GSVA results for the DEGs, which were enriched in the resistant group or the sensitive group (highlighted orange points and blue points, respectively). DEGs: Differentially expressed genes. GSEA: Gene Set Enrichment Analysis. GSVA: Gene Set Variation Analysis

GSEA and GSVA of the DEGs (Tables 3 and 4, respectively) indicated that the downregulated genes were enriched in “HALLMARK_COMPLEMENT” (Fig. 3a), “HALLMARK_BILE_ACID_METABOLISM” (Fig. 3b), “HALLMARK_IL2_STATS_SIGNALING” (Fig. 3c), “HALLMARK_UV_RESPONSE_DN” (Fig. 3d), “HALLMARK_ESTROGEN_RESPONSE_EARLY” (Fig. 3e), “HALLMARK_APOPTOSIS” (Fig. 3f), “HALLMARK_INFLAMMATORY_RESPONSE” (Fig. 3g), and “HALLMARK_ESTROGEN_RESPONSE_LATE” (Fig. 3h). The upregulated genes were enriched in “HALLMARK_PEROXISOME” (Fig. 3i). The GSVA results suggested that the downregulated genes were enriched in “HALLMARK_MYC_TARGETS_V1 and V2” and upregulated genes were enriched in “HALLMARK_MYOGENESIS,” “HALLMARK_ANGIOGE-NESIS,” “HALLMARK_INTERFERON_ALPHA_RESPONSE,” and “HALLMARK_TGF_ BETA_SIGNALING” (Fig. 3j).

**Table 3:** Gene set enrichment analysis of upregulated and downregulated gene sets in the cetuximab resistant group

Name		Size	Es	Nes		Non *p* value	FDR *q* value		FWER *p* value	
Up-regulation in Resistant Group										
HALLMARK_PEROXISOME		19	−0.36	−1.32		0.00	0.49		0.39	
HALLMARK_UV_RESPONSE_UP		24	−0.17	−1.00		0.38	1.00		1.00	
HALLMARK_MYOGENESIS		38	−0.48	−0.93		0.79	1.00		1.00	
HALLMARK_KRAS_SIGNALING_DN		27	−0.24	−0.92		0.54	1.00		1.00	
HALLMARK_ADIPOGENESIS		24	−0.17	−0.80		0.51	1.00		1.00	
HALLMARK_INTERFERON_GAMMA_RESPONSE		31	−0.21	−0.77		0.91	1.00		1.00	
HALLMARK_INTERFERON_ALPHA_RESPONSE		24	−0.28	−0.76		0.91	0.98		1.00	
HALLMARK_P53_PATHWAY		24	−0.14	−0.68		0.91	0.92		1.00	
Down-regulation in Resistant Group										
HALLMARK_COMPLEMENT		30	0.34	1.59		0.00	0.27		0.09	
HALLMARK_BILE_ACID_METABOLISM		21	0.38	1.57		0.00	0.19		0.19	
HALLMARK_IL2_STAT5_SIGNALING		31	0.38	1.50		0.00	0.16		0.25	
HALLMARK_UV_RESPONSE_DN		24	0.42	1.47		0.00	0.13		0.25	
HALLMARK_ESTROGEN_RESPONSE_EARLY		39	0.30	1.43		0.00	0.12		0.25	
HALLMARK_HYPOXIA		38	0.30	1.41		0.09	0.10		0.25	
HALLMARK_APOPTOSIS		30	0.28	1.35		0.00	0.19		0.43	
HALLMARK_INFLAMMATORY_RESPONSE		29	0.44	1.29		0.00	0.25		0.56	
HALLMARK_GLYCOLYSIS		16	0.39	1.22		0.32	0.34		0.56	
HALLMARK_ESTROGEN_RESPONSE_LATE		34	0.40	1.21		0.00	0.31		0.56	
HALLMARK_XENOBIOTIC_METABOLISM		30	0.25	1.20		0.09	0.31		0.62	
HALLMARK_KRAS_SIGNALING_UP		41	0.48	1.13		0.32	0.48		0.67	
HALLMARK_TNFA_SIGNALING_VIA_NFKB		30	0.32	1.13		0.34	0.45		0.67	
HALLMARK_HEME_METABOLISM		21	0.29	1.08		0.29	0.59		0.95	
HALLMARK_APICAL_JUNCTION		26	0.28	0.99		0.44	0.88		1.00	
HALLMARK_COAGULATION		30	0.26	0.99		0.45	0.85		1.00	
HALLMARK_G2M_CHECKPOINT		19	0.40	0.98		0.54	0.82		1.00	
HALLMARK_E2F_TARGETS		21	0.37	0.97		0.69	0.79		1.00	
HALLMARK_MITOTIC_SPINDLE		15	0.29	0.95		0.66	0.80		1.00	
HALLMARK_MYC_TARGETS_V1		21	0.37	0.93		0.79	0.80		1.00	
HALLMARK_OXIDATIVE_PHOSPHORYLATION		17	0.32	0.91		0.82	0.83		1.00	
HALLMARK_EPITHELIAL_MESENCHYMAL_TRANSITION		44	0.29	0.91		0.59	0.80		1.00	
HALLMARK_IL6_JAK_STAT3_SIGNALING		16	0.27	0.87		0.53	0.84		1.00	
HALLMARK_ALLOGRAFT_REJECTION		22	0.23	0.81		0.75	0.89		1.00	
HALLMARK_MTORC1_SIGNALING		19	0.19	0.67		0.91	0.96		1.00	
HALLMARK_FATTY_ACID_METABOLISM		31	0.14	0.61		0.91	0.95		1.00	

**Table 4:** Gene set variation analysis of upregulated and downregulated gene sets in the cetuximab resistant group

HALLMARK GENE SETS	Log [FC]	*p* value	Adjustive *p* value
HALLMARK_MYOGENESIS	1.058	2.34E−05	3.89E−04
HALLMARK_INTERFERON_ALPHA_RESPONSE	1.115	3.21E−05	3.89E−04
HALLMARK_MYC_TARGETS_V1	−1.096	3.89E−05	3.89E−04
HALLMARK_MYC_TARGETS_V2	−1.134	2.88E−05	3.89E−04
HALLMARK_ANGIOGENESIS	1.229	1.43E−05	3.89E−04
HALLMARK_TGF_BETA_SIGNALING	1.078	9.87E−05	6.17E−04
HALLMARK_INTERFERON_GAMMA_RESPONSE	0.909	9.74E−05	6.17E−04
HALLMARK_UNFOLDED_PROTEIN_RESPONSE	−0.978	7.86E−05	6.17E−04
HALLMARK_E2F_TARGETS	−0.915	2.91E−04	1.45E−03
HALLMARK_EPITHELIAL_MESENCHYMAL_TRANSITION	0.741	2.72E−04	1.45E−03
HALLMARK_APICAL_JUNCTION	0.820	3.21E−04	1.46E−03
HALLMARK_G2M_CHECKPOINT	−0.926	7.41E−04	3.09E−03
HALLMARK_APOPTOSIS	0.574	1.13E−03	4.13E−03
HALLMARK_HEME_METABOLISM	0.610	1.16E−03	4.13E−03
HALLMARK_KRAS_SIGNALING_UP	−0.622	1.24E−03	4.13E−03
HALLMARK_ESTROGEN_RESPONSE_EARLY	0.572	1.41E−03	4.16E−03
HALLMARK_PEROXISOME	0.676	1.38E−03	4.16E−03
HALLMARK_UV_RESPONSE_DN	0.624	1.95E−03	5.42E−03
HALLMARK_HEDGEHOG_SIGNALING	0.742	1.96E−02	4.97E−02
HALLMARK_OXIDATIVE_PHOSPHORYLATION	−0.599	2.09E−02	4.97E−02
HALLMARK_REACTIVE_OXYGEN_SPECIES_PATHWAY	0.706	2.03E−02	4.97E−02

### Common DEGs and PPI network construction

**Fig. 4:**
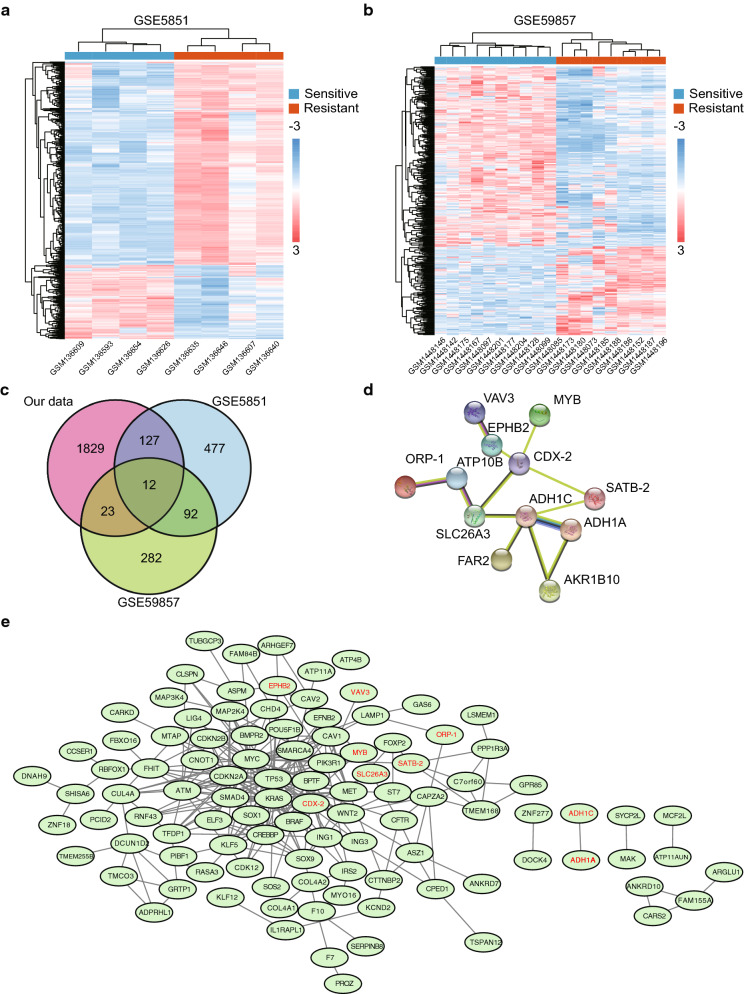
Identification of common DEGs and construction of PPI network. **a**,** b** Identification of DEGs between the resistant (red) and sensitive (blue) groups from the GSE5851 and GSE59857 datasets, illustrated by a heat map. Each column is a sample and each row is a gene. **c** The venn diagram shows the intersection of DEGs among GSE5851, GSE59857 and CACO2-CR cellular model. **d** The Network diagram illustrates the interactions of common DEGs. **e** The PPI network including 12 DEGs and recognized cetuximab resistance-related genes from the Genomics of Drug Sensitivity in Cancer database. DEGs: Differentially expressed genes. PPI: protein-protein interaction network

**Fig. 5:**
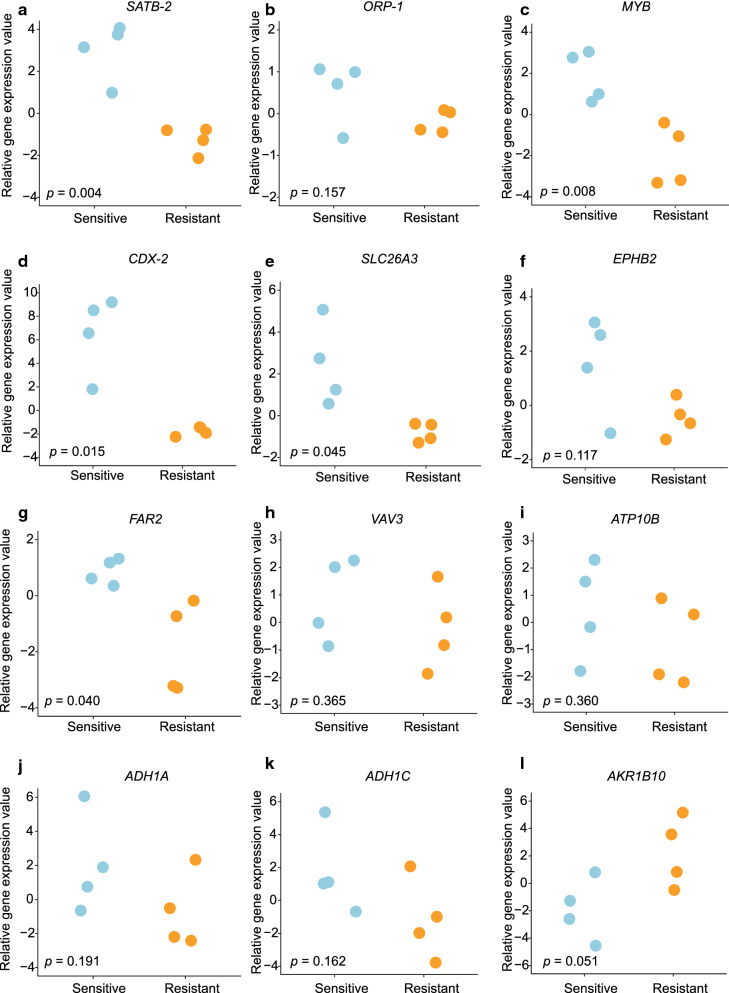
Comparison of the expression levels of core DEGs in GSE56386. **a ***SATB-2*, **c ***MYB*, **d ***CDX-2*, **e ***SLC26A3*, and **g ***FAR2* were significantly downregulated and (**l**)*AKR1B10* was apparently upregulated among cetuximab non-responders compared with cetuximab responders. No significant differences were seen in (**b**)*ORP-1*, **f ***EPHB2*, **h ***VAV3*, **i ***ATP10B*, **j ***ADH1A*, **k ***ADH1C*. DEGs: Differentially expressed genes

We identified 708 (Fig. 4a) and 298 (Fig. 4b) DEGs in GSE5851 and GSE59857, respectively. After integrating the DEGs from our own screenings with those from the two GEO datasets, 12 common DEGs were identified (Fig. 4c). These DEGs did not represent any protein-level interactions (Fig. 4d). Then, we combined recognized cetuximab resistance-related genes from the GDSC database (Table 5) with the 12 common DEGs and constructed a PPI network (Fig. 4e). The following six core DEGs were identified: *SATB-2*, *ORP-1, MYB*, *CDX-2*, *SLC26A3*, and *EPHB2*. Consequently, we selected the GSE56386 dataset to validate the variations in the expression levels of the 12 DEGs between cetuximab responders and non-responders in clinical terms. *SATB-2*, *MYB*, *CDX-2*, *SLC26A3*, and *FAR2* were downregulated (Fig. 5a, c–e, g) and *AKR1B10* was upregulated in the non-responder group (Fig. 5l). Although a trend of downregulation was observed in the other six genes (Fig. 5b, f, h–k), they exhibited no significant differences between two groups, possibly owing to the small sample size. These 12 genes had an optimal prediction accuracy in GSE56386. The receiver operating characteristic curves for the 12 genes are presented in Additional file 2: Fig. S1.

**Table 5:** Cetuximab resistance-related genes from the Genomics of Drug Sensitivity in Cancer database

Cancer feature	Effect size	*p* value	FDR%	No. of altered cell lines	Tissue analysis
SRGAP3_mut	−1.310	0.0382	99.7	3	COREAD
PBRM1_mut	−0.971	0.0791	99.7	4	COREAD
B2M_mut	−0.722	0.0879	99.7	7	COREAD
FBXW7_mut	−0.575	0.0998	99.7	13	COREAD
BRAF_mut	0.580	0.109	99.7	10	COREAD
cnaCOREAD47	0.737	0.111	99.7	6	COREAD
cnaCOREAD18	0.611	0.124	99.7	8	COREAD
MGA_mut	−0.688	0.132	99.7	6	COREAD
cnaCOREAD14	0.892	0.147	99.7	3	COREAD
CTCF_mut	−0.560	0.17	99.7	7	COREAD
cnaCOREAD37	0.634	0.172	99.7	6	COREAD
CDH1_mut	−0.819	0.195	99.7	3	COREAD
CTNNB1_mut	−0.577	0.199	99.7	6	COREAD
CREBBP_mut	0.530	0.213	99.7	6	COREAD
CHD9_mut	−0.468	0.231	99.7	9	COREAD
BRWD1_mut	−0.740	0.231	99.7	3	COREAD
CHD4_mut	0.518	0.285	99.7	3	COREAD
APC_mut	−0.285	0.313	99.7	31	COREAD
AKAP9_mut	−0.412	0.316	99.7	8	COREAD
cnaCOREAD32	−0.372	0.347	99.7	6	COREAD

### Core DEGs and tumor immune cell infiltration

**Fig. 6:**
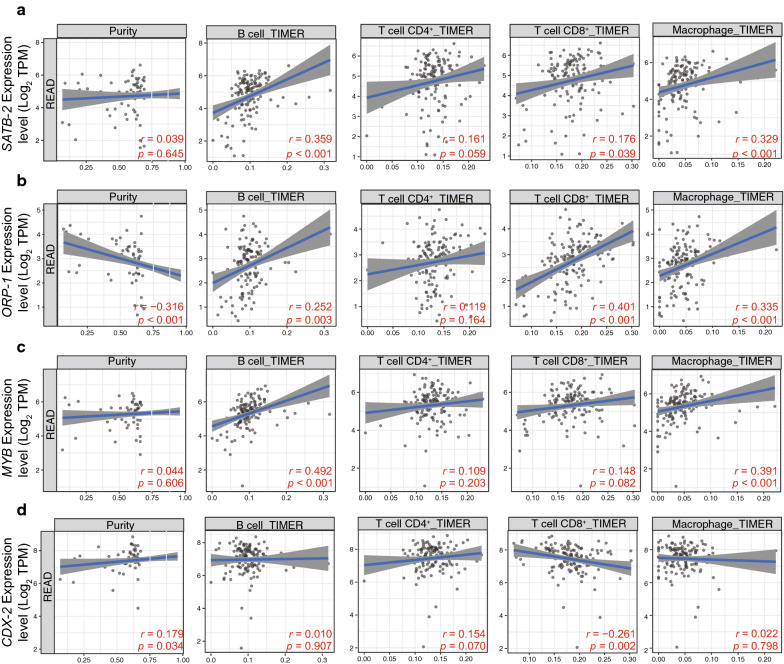
Association between the expression level of common DEGs and immune cell infiltration. The relationship between expression levels of (**a**) *SATB-2*, (**b**) *ORP-1*, (**c**) *MYB*, and (**d**) *CDX-2* and tumor purity, infiltrating B cells, CD4^+^ T cells, CD8^+^ T cells, and macrophages was investigated by the online tool Tumor Immune Estimation Resource Version. DEGs: Differentially expressed genes. TIMER: Tumor Immune Estimation Resource Version

**Fig. 7:**
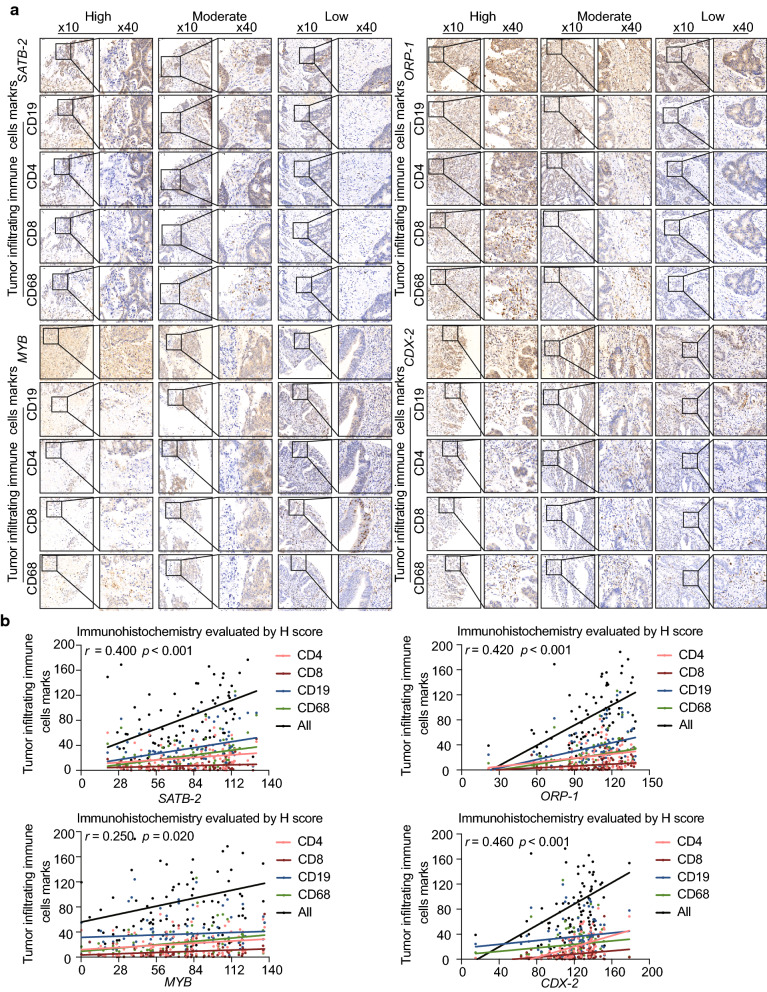
Relationship between core DEGs and tumor-infiltrating immune cell markers in CRC. **a** The representative immunohistochemical staining images of *SATB-2*, *ORP-1*, *MYB*, *CDX-2*, CD19, CD4, CD8, and CD68. **b** Spearman analysis results indicated that core DEGs were positively associated with tumor-infiltrating immune cell markers. DEGs: Differentially expressed genes. CRC: Colorectal cancer

We observed significant correlations between the expression levels of the core DEGs *SATB-2* (Fig. 6a), *ORP-1* (Fig. 6b), *MYB* (Fig. 6c), and *CDX-2* (Fig. 6d) and tumor immune cell infiltration represented by the expression of B cell, CD4^+^ T cell, CD8^+^ T cell, and macrophage markers in TCGA. Immunohistochemical staining of a tissue microarray containing 102 CRC clinical samples (Fig. 7a) demonstrated that the expression of these four genes was positively associated with tumor-infiltrating immune cell markers such as CD4, CD8, CD19, and CD68 (Fig. 7b; Table 6).

**Table 6:** Spearman correlation analysis of differentially expressed genes and tumor-infiltrating immune cell markers in clinical samples

Tumor-infiltrating immune cells marks	Gene expression level		
	Spearman correlation	95% CI	*p* value
*SATB-*			
CD4	0.22	0.00 to 0.42	0.047
CD8	0.15	-0.07 to 0.35	0.184
CD19	0.36	0.16 to 0.54	< 0.001
CD68	0.34	0.14 to 0.52	0.002
CD4 and CD8	0.22	0.07 to 0.42	0.044
CD4, CD8, CD19, CD68	0.40	0.20 to 0.57	< 0.001
*ORP-1*			
CD4	0.28	0.07 to 0.46	0.010
CD8	0.24	0.03 to 0.43	0.030
CD19	0.36	0.16 to 0.53	< 0.001
CD68	0.31	0.11 to 0.50	0.003
CD4 and CD8	0.29	0.08 to 0.48	0.007
CD4, CD8, CD19, CD68	0.42	0.23 to 0.58	< 0.001
*MYB*			
CD4	0.22	0.01 to 0.42	0.040
CD8	0.20	-0.01 to 0.40	0.070
CD19	0.09	-0.13 to 0.30	0.450
CD68	0.28	0.07 to 0.47	0.009
CD4 and CD8	0.26	0.05 to 0.45	0.020
CD4, CD8, CD19, CD68	0.25	0.04 to 0.44	0.020
*CDX-2*			
CD4	0.66	0.52 to 0.77	< 0.001
CD8	0.35	0.14 to 0.53	0.001
CD19	0.20	-0.02 to 0.40	0.070
CD68	0.23	0.01 to 0.43	0.030
CD4 and CD8	0.65	0.50 to 0.76	< 0.001
CD4, CD8, CD19, CD68	0.46	0.27 to 0.62	< 0.001

### **Exploring the expression of identified core DEGs** in vitro

**Fig. 8:**
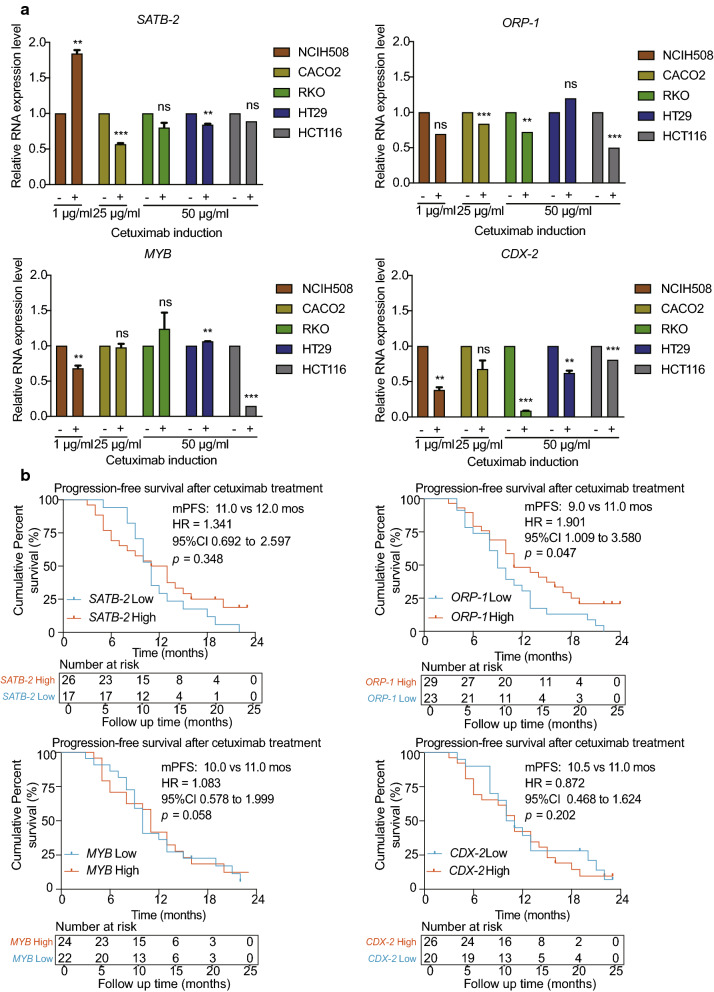
Exploring the expression of core DEGs in vitro and their prognostic roles in CRC. **a** *SATB-2*, *ORP-1*, *MYB*, and *CDX-2* were downregulated after cetuximab treatment. **b** Patients with high expression levels of core DEGs in primary tumors exhibited a tendency to experience a longer progression-free survival time. **, *p* < 0.01, ***, *p* < 0.001, ns, *p* > 0.05. DEGs: Differentially expressed genes. CRC: Colorectal cancer

We conducted preliminary experiments in several CRC cell lines and detected that *ORP-1*, *MYB*, and *CDX-2* were downregulated in NCIH508^*wtRAS/RAF*^ (a cell line sensitive to cetuximab), while *SATB-2* expression decreased in CACO2 ^*wtRAS/RAF*^ (a cell line partially sensitive to cetuximab) and HT29 ^*wtRAS/mtRAF*^ (a cell line resistant to cetuximab) at the half-maximal inhibition concentration of cetuximab. A reduction in *ORP-1* and *CDX-2* expression was observed in most of these cell lines regardless of *RAS* and *RAF* status (Fig. 8a).

### The roles of identified core DEGs in clinical samples with anti-EGFR therapy

The clinicopathological characteristics of the patients were recorded and are shown in Additional file 3: Table S2. Median follow-up time was 33.1 months (interquartile range, 17.2 to 52.5 months). Compared with patients with low *ORP-1* expression, those with high *ORP-1* expression in CRC experienced a significantly longer PFS time (median times were 9.0 months and 11.0 months, respectively, hazard rate [HR] = 1.901, *p* = 0.047) after administration of chemotherapeutics containing cetuximab (Fig. 8b).

## Discussion

Alterations in genes that function in the epidermal growth factor (EGF) signaling pathway, such as increases in *EGFR* copy number, amplification of *ERBB* family, overexpression of *IGF1* or *VEGF*, or novel mutations such as point mutations in *RAS*, *BRAF*, *PI3KCA*, or *MEK* in the EGFR extracellular domain or in the downstream pathway, result in EGFR antagonist resistance. Recently, disturbances in the tumor microenvironment caused by EGFR antibody have also been recognized as factors in treatment failure. Garvey et al. reported that cetuximab causes cancer-associated fibroblasts to secrete more EGF and reactivate mitogen-activated protein kinase signaling in para-CRC cells [32]. Critically, it is important to understand the interaction between tumor cells and the tumor microenvironment in anti-EGFR therapy resistance.

We conducted an integrated bioinformatic analysis to identify nine common DEGs between the cetuximab sensitive and resistant groups combining high-throughput data of the cetuximab resistant cell line model and data of cell lines and clinical samples from GEO profiles. The relationship between DEGs identified in our study and tumor immune cell infiltration was evaluated in TCGA and validated in clinical samples from our hospital. We found that four (*SATB-2*, *ORP-1*, *MYB*, and *CDX-2*) of nine DEGs were associated with infiltrated T cells, B cells, and macrophages in CRC. The decreasing trend of expression levels of *ORP-1*, *MYB* and *CDX-2* under cetuximab pressure was observed in vitro (Additional file 4: Fig. S2), which was consistent with the expression changes in the established cetuximab resistant cell line (CACO2-CR). Moreover, patients with high expression levels of these genes, especially *ORP-1*, exhibited prolonged survival receiving anti-EGFR therapy.

*SATB-2* encodes a DNA binding protein and mediates transcription regulation as well as chromatin remodeling. *SATB-2* attenuates the activity of MEK5/ERK5 and suppresses tumorigenesis and metastasis [33]. The upregulation of *SATB-2* via DNA demethylation of the promoter region and H3K4me3 increases TH1-type chemokine expression and immune cell density in CRC [34]. *ORP-1*, as a member of the oxysterol-binding protein family involve in human innate immune system, binds to phosphatidylinositol 3-phosphate by interacting with *RAB7A* and stabilizing GTP-RAB7A and regulates the MHC class I -mediated antigen processing and presentation pathway [35, 36]. In addition, *ORP-1* suppresses tumorigenesis via metabolism-associated pathway [37]. *MYB* is a protein-encoding oncogene that functions as a transcriptional activator. Paradoxically, patients with CRC and high *MYB* expression exhibit low incidence of distant metastases [38] and favorable clinical prognosis [39]. Millen et al. demonstrated that a high level of CD8^+^ tumor immune infiltrating cells and a clinical history of longer relapse-free survival were related to high expression of *MYB*. An immunomodulatory effect conferred by *MYB* was also observed in CD8^+^ TILs in the murine CRC model [40]. *CDX-2* encodes a regulator of intestine-specific genes involved in cell growth and differentiation. *CDX-2* is downregulated in the invasive part of tumor tissues and is associated with tumor-stroma protein expression as well as inflammatory cytokine release in CRC [41]. Low levels of *CDX-2* expression indicate a particularly poor survival prognosis, especially in patients with tumors that have a high stromal content [42].

Zanella et al. reported that EGFR antagonists inhibit colorectal tumor growth and simultaneously protect the tumor from inhibition by transcriptional regulation [43]. Woolston et al. supported this finding and revealed that cetuximab prompts a transformation from a mutational variation to a mesenchymal transition representative of tumor-associated fibroblast enrichment [20]. It seems that cetuximab evokes immune inflammation via antibody-dependent cell-mediated cytotoxicity but paradoxically weakens this effect via a form of IgG1 antibody-mediated immunogenic cell death [21] while upregulating immunosuppressive TGF-β expression in CRC [44]. In fact, in a stage Ib/II trial of combined cetuximab and pembrolizumab treatment for patients with advanced CRC, the combination treatment of cetuximab with pembrolizumab significantly increased the density of CD3^+^, CD8^+^, and CTLA-4^+^ lymphocytes and natural killer cells in tumors. In the peripheral blood, the overall density of CD4^+^ and CD8^+^ lymphocytes decrease, especially that of the PD1^+^ memory T cells [45, 46]. In the process of acquiring secondary resistance, the tumor might experience the transition from the immune-inflamed phenotype to the immune-desert phenotype, which features by key target genes involved in tumor immune cell infiltration [47–49]. We believed that the core DEGs identified in this study, *SATB-2*, *ORP-1*, *MYB*, and *CDX-2*, might play critical roles in the transition.

Nevertheless, there were some limitations lying in this study. As models of anti-EGFR antibody resistance are not easy to established, only one cellular model was established and screened in the study. More cetuximab resistant cellular models with wild-type *RAS* and *RAF* genotypes are required to be established for validation. The DEGs identified using in silico methods should be further validated in vitro and in vivo*.* The results drawn from the retrospective clinical cohort with a small sample size also need further verification in a larger population. The specific immune cell types involved in secondary anti-EGFR antibody resistance should be more clearly elucidated.

## Conclusions

In summary, we distinguished cetuximab-induced DEGs associated with variations of tumor immune cell infiltration in CRC by establishing a cetuximab resistant cellular model and integrated bioinformatics analysis. The results suggested that transcriptomic alterations and immune landscape remodeling should receive additional scrutiny during anti-EGFR antibody treatment. Furthermore, immunotherapy could be considered in the early stages of cetuximab treatment rather than after resistance has already occurred.

## Supplementary Information

**Additional file 1: Table S1.** Sequences of primers used for real-time quantitative PCR.

**Additional file 2: Figure S1.** ROC curves for the cetuximab sensitivity of the 12 DEGs in GSE56386. DEGs: Differentially expressed genes. ROC: Receiver operating characteristic.

**Additional file 3: Table S2.** Clinicopathological data of patients with colorectal cancer in the tissue microarray.

**Additional file 4: Figure S2.** *SATB-2*, *ORP-1*, *MYB*, and *CDX-2* were downregulated in the resistant CACO2. ##, *p* < 0.01, ###, *p* < 0.001.

## Data Availability

Public datasets of colon adenocarcinoma and rectum adenocarcinoma analyzed during the current study can be retrieved from The Cancer Genome Atlas (TCGA) at https://portal.gdc.cancer.gov/. GSE5851, GSE56368, and GSE59857 datasets can be retrieved from the Gene Expression Omnibus (GEO) at https://www.ncbi.nlm.nih.gov/geo/. Other data that support the findings of this study are available from the corresponding author on reasonable request.

## Abbreviations

CRC: Colorectal cancer
EGFR: Epidermal growth factor receptor
GEO: Gene Expression Omnibus
DEGs: Differentially expressed genes
PCA: Principal component analysis
GO: Gene Ontology
KEGG: Kyoto Encyclopedia of Genes and Genomes
GSEA: Gene Sets Enrichment Analysis
GSVA: Gene Set Variation Analysis
PPI: Protein-protein interaction
GDSC: Genomics of Drug Sensitivity in Cancer
TCGA: The Cancer Genome Atlas
OSBP: Oxysterol-binding protein
TILs: Tumor immune infiltrating cells
COAD: Colon adenocarcinoma
READ: Rectum adenocarcinoma
