# Supplementary material for: Identification of key genes involved in tumor immune cell infiltration and cetuximab resistance in colorectal cancer
Source: Cancer Cell Int. 2021 Feb 25;21:135. doi: 10.1186/s12935-021-01829-8 (PMC7905896; doi:10.1186/s12935-021-01829-8)
Supplement: Supplementary file 1 — Additional file 1: Table S1. Sequences of primers used for real-time quantitative PCR. [file 12935_2021_1829_MOESM1_ESM.docx]

**Table S1 Sequences of primers used for real-time quantitative PCR.**

| **Primers** | **Sequence (5’-3’)** |
| --- | --- |
| *CDX-2*-F | GGTTTCAGAACCGCAGAGCA |
| *CDX-2*-R | CAAGGGCTCTGGGACACTTC |
| *MYB*-F | CATGTTCCATACCCTGTAGCG |
| *MYB*-R | TTCTCGGTTGACATTAGGAGC |
| *ORP-1*-F | GAAGAGTGTAGAAGCAGAACCC |
| *ORP-1*-R | TGCCATACTGTTCGATCCAC |
| *SATB-2*-F | CTGCGTCTTCTCGGCTCTTG |
| *SATB-2*-R | CGTTCTGGAGAGAAAGGGCTG |
| *GAPDH*-F | AGCCACATCGCTCAGACAC |
| *GAPDH*-R | GCCCAATACGACCAAATCC |
