# Supplementary figures and images for: Identification of key genes involved in tumor immune cell infiltration and cetuximab resistance in colorectal cancer
Source: Cancer Cell Int. 2021 Feb 25;21:135. doi: 10.1186/s12935-021-01829-8 (PMC7905896; doi:10.1186/s12935-021-01829-8)

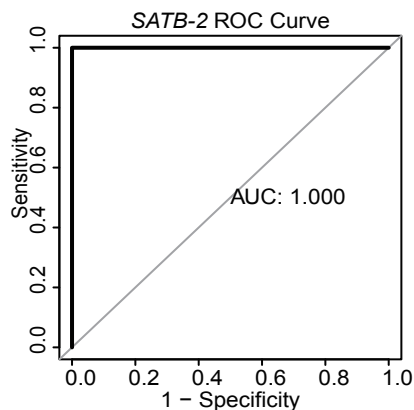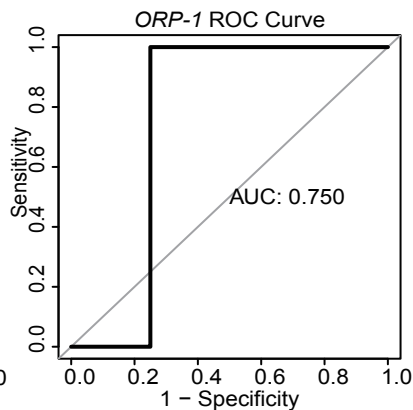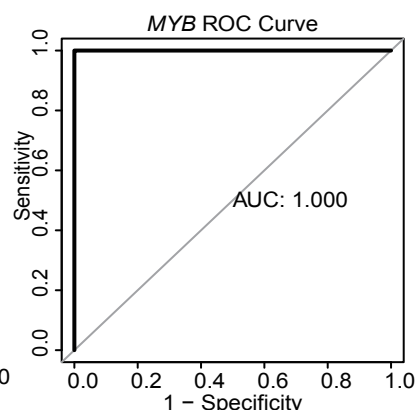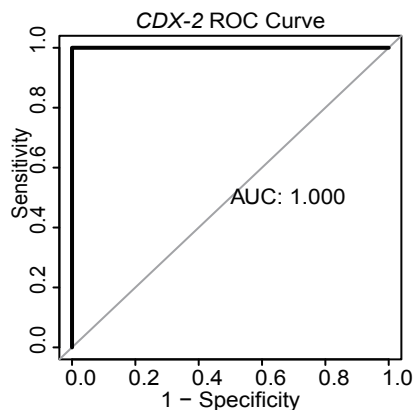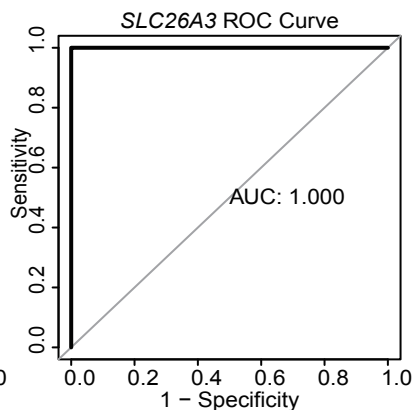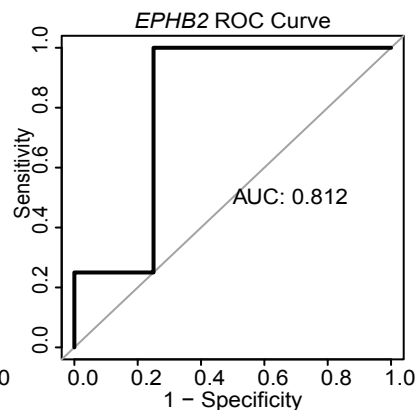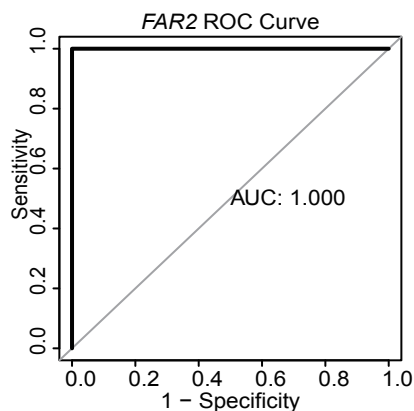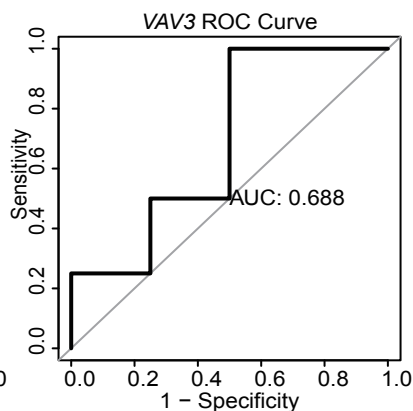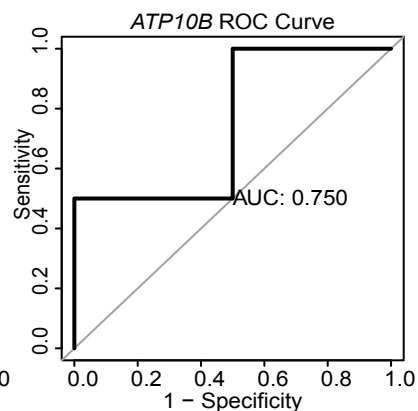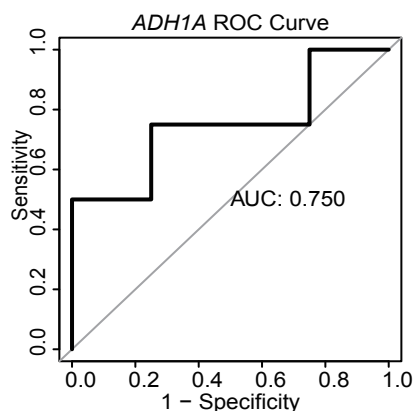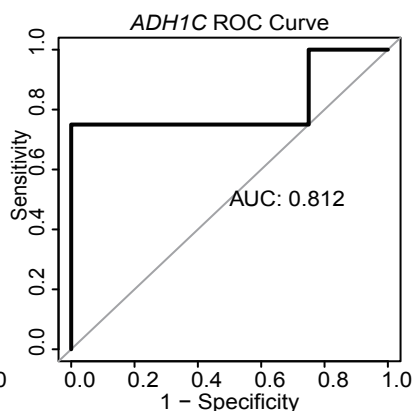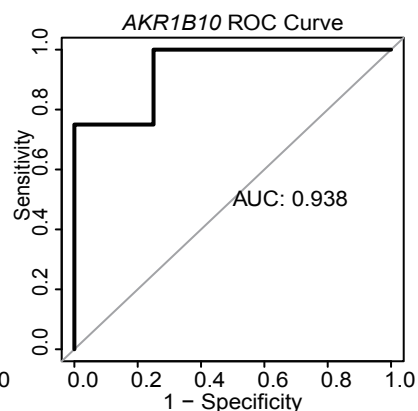

Supplement: Supplementary file 2 — Additional file 2: Figure S1. ROC curves for the cetuximab sensitivity of the 12 DEGs in GSE56386. DEGs: Differentially expressed genes. ROC: Receiver operating characteristic. [file 12935_2021_1829_MOESM2_ESM.pdf]

# CACO2

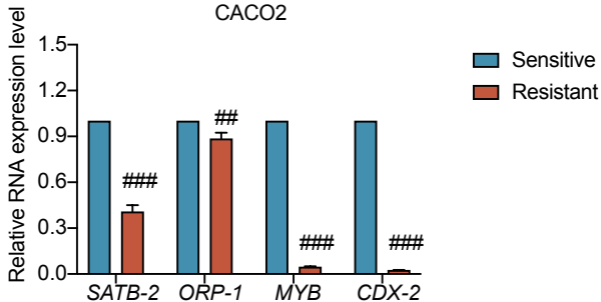

Supplement: Supplementary file 4 — Additional file 4: Figure S2. SATB-2, ORP-1, MYB, and CDX-2 were downregulated in the resistant CACO2. ##, p < 0.01, ###, p < 0.001. [file 12935_2021_1829_MOESM4_ESM.pdf]
