# Supplementary material for: Identification of key genes involved in tumor immune cell infiltration and cetuximab resistance in colorectal cancer
Source: Cancer Cell Int. 2021 Feb 25;21:135. doi: 10.1186/s12935-021-01829-8 (PMC7905896; doi:10.1186/s12935-021-01829-8)
Supplement: Supplementary file 3 — Additional file 3: Table S2. Clinicopathological data of patients with colorectal cancer in the tissue microarray. [file 12935_2021_1829_MOESM3_ESM.docx]

**Table S2 Clinicopathological data of patients with colorectal cancer in the tissue microarray.**

| **Clinic pathological characteristics** | **Number of Cases/Value** | **%** |
| --- | --- | --- |
| Primary tumor site |  |  |
| Colon | 62 | 60.78 |
| Rectum | 40 | 39.22 |
| Colon location |  |  |
| Right | 30 | 29.41 |
| Left | 72 | 70.59 |
| Gender |  |  |
| Male | 71 | 69.61 |
| Female | 31 | 30.39 |
| Age |  |  |
| less than 60 | 56 | 54.90 |
| more than 60 | 46 | 45.10 |
| Initial stage |  |  |
| Advanced | 53 | 51.96 |
| Recurrence | 49 | 48.04 |
| Survival statues |  |  |
| Death | 48 | 47.06 |
| Alive | 54 | 52.94 |
| Primary tumor resection |  |  |
| Radical | 53 | 51.96 |
| Palliative | 49 | 48.04 |
| Anti-EGFR therapy |  |  |
| Yes | 52 | 50.98 |
| No | 50 | 49.02 |
| Success staining |  |  |
| *SATB-2* | 96 | 94.12 |
| *OPR-1* | 94 | 92.16 |
| *MYB* | 90 | 88.24 |
| *CDX-2* | 92 | 90.20 |
| CD19 | 94 | 92.16 |
| CD4 | 96 | 94.12 |
| CD8 | 98 | 96.08 |
| CD68 | 101 | 99.02 |
| Average H score |  |  |
| *SATB-2* | 80.19 | / |
| *OPR-1* | 104.57 | / |
| *MYB* | 71.19 | / |
| *CDX-2* | 117.77 | / |
| CD19 | 37.95 | / |
| CD4 | 20.30 | / |
| CD8 | 8.98 | / |
| CD68 | 23.54 | / |
| Median H score |  |  |
| *SATB-2* | 83.38 | / |
| *OPR-1* | 111.05 | / |
| *MYB* | 70.77 | / |
| *CDX-2* | 124.18 | / |
| CD19 | 29.93 | / |
| CD4 | 14.43 | / |
| CD8 | 3.98 | / |
| CD68 | 16.41 | / |
| Total | 102 | 100.00 |
